# Prediction of chemotherapy benefit by EndoPredict in patients with breast cancer who received adjuvant endocrine therapy plus chemotherapy or endocrine therapy alone

**DOI:** 10.1007/s10549-019-05226-8

**Published:** 2019-04-30

**Authors:** Ivana Sestak, Miguel Martín, Peter Dubsky, Ralf Kronenwett, Federico Rojo, Jack Cuzick, Martin Filipits, Amparo Ruiz, William Gradishar, Hatem Soliman, Lee Schwartzberg, Richard Buus, Dominik Hlauschek, Alvaro Rodríguez-Lescure, Michael Gnant

**Affiliations:** 1 Centre for Cancer Prevention, Wolfson Institute of Preventive Medicine, Queen Mary University of London, Charterhouse Square, London, EC1M 6BQ UK; 2 Instituto de Investigacion Sanitaria Gregorio Marañon, CIBERONC, Universidad Complutense, Madrid, Spain; 3 Spanish Breast Cancer Group, GEICAM, Madrid, Spain; 4 Hirslanden Klinik St. Anna, Lucerne, Switzerland; 5 Department of Surgery and Comprehensive Cancer Center, Medical University of Vienna, Vienna, Austria; 6 Myriad International GmbH, Cologne, Germany; 7 Fundacion Jimenez Diaz, Madrid, Spain; 8 Austrian Breast and Colorectal Study Group, ABCSG, Vienna, Austria; 9 Instituto Valenciano de Oncologia, Valencia, Spain; 10 Robert H. Lurie Comprehensive Cancer Center of Northwestern University, Chicago, USA; 11 H. Lee Moffitt Cancer Center and Research Institute, Tampa, FL USA; 12 West Cancer Center, Germantown, USA; 13 The Breast Cancer Now Research Centre, Institute of Cancer, London, UK; 14 Ralph Lauren Centre for Breast Cancer Research, Royal Marsden Hospital, London, UK; 15 Hospital Universitario de Elche, Valencia, Spain

**Keywords:** Chemotherapy, Prediction, Breast cancer, EndoPredict

## Abstract

**Purpose:**

EndoPredict (EPclin) is a prognostic test validated to inform decisions on adjuvant chemotherapy to endocrine therapy alone for patients with oestrogen receptor-positive, HER2-negative breast cancer. Here, we determine the performance of EPclin for estimating 10-year distant recurrence-free interval (DRFI) rates for those who received adjuvant endocrine therapy (ET) alone compared to those with chemotherapy plus endocrine therapy (ET + C).

**Methods:**

A total of 3746 women were included in this joint analysis. 2630 patients received 5 years of ET alone (ABCSG-6/8, TransATAC) and 1116 patients received ET + C (GEICAM 2003-02/9906). The primary objective was to evaluate the ability of EPclin to provide an estimate of the 10-year DR rate as a continuous function of EPclin separately for ET alone and ET + C. Cox proportional hazard models were used for these analyses.

**Results:**

EPclin was highly prognostic for DR in women who received ET alone (HR 2.79 (2.49–3.13), *P* < 0.0001) as well as in those who received ET + C (HR 2.27 (1.99–2.59), *P *< 0.0001). Women who received ET + C had significantly smaller increases in 10-year DR rates with the increasing EPclin score than those receiving ET alone (EPclin = 5; 12% ET + C vs. 20% ET alone). We observed a significant positive interaction between EPclin and treatment groups (*P*-_interaction_ = 0.022).

**Conclusions:**

In this comparative non-randomised analysis, the rate of increase in DR with EPclin score was significantly reduced in women who received ET + C versus ET alone. Our indirect comparisons suggest that a high EPclin score can predict chemotherapy benefit in women with ER-positive, HER2-negative disease.

**Electronic supplementary material:**

The online version of this article (10.1007/s10549-019-05226-8) contains supplementary material, which is available to authorized users.

## Introduction

For the clinical management of patients with oestrogen receptor (ER)-positive breast cancer, several clinico-pathological and molecular characteristics of the tumour have to be considered for prognosis and treatment decisions. Almost all women with ER-positive, HER2-negative breast cancer will receive at least 5 years of endocrine therapy but the question who will benefit from adjuvant chemotherapy is more challenging. Comparisons of different adjuvant polychemotherapy regimens have shown that breast cancer mortality can be reduced by about one-third, but this reduction depends on absolute risks without chemotherapy and proportional reductions were largely independent by clinico-pathological parameters [1]. Over the last two decades several multigene tests have been developed to aid the selection of patients for whom adjuvant chemotherapy might be appropriate based on prognosis [2, 3]. All of these tests predict the likelihood of disease recurrence or progression [2, 4–7], and some have shown to predict relative benefit from chemotherapy [8, 9]. However, none of the multigene tests have so far shown that they can aid in the decision-making process regarding which chemotherapy regime to use [10, 11].

The EndoPredict test combines the expression of three proliferative and five ER-signalling-associated genes together with four normalisation and control genes and can be measured in formalin-fixed, paraffin-embedded tissue sections by quantitative real-time polymerase chain reaction (qRT-PCR) in decentralised laboratories [12]. EPclin incorporates information on nodal status and tumour size and is used as the diagnostic algorithm in the clinical setting. EPclin has been validated as a prognostic test in pre- and postmenopausal women with ER-positive, HER2-negative breast cancer [6, 11, 13, 14]. EPclin was highly prognostic and identified a large proportion of women as low risk with a less than ten percent 10-year risk of distant recurrence [6, 14]. In the TransATAC cohort, the two signatures that include clinicopathological parameters, EPclin and Prosigna, were substantially more prognostic and had a superior risk stratification when compared to immunohistochemical markers, Oncotype Dx Recurrence score, or Breast Cancer Index, particularly in women with lymph node-positive disease who are most likely to receive chemotherapy [2]. In the GEICAM/9906 trial, EPclin identified a group of patients with node-positive disease who had a particularly low risk of distant recurrence and an absolute risk reduction of 30% compared to patients with high-risk disease [15].

Retrospective and prospective clinical trials looking at chemotherapy prediction using multigene tests in women with lymph node-negative or node-positive disease have reported their results [8, 9, 16, 17]. Oncotype Dx Recurrence score was shown to predict chemotherapy benefit, mostly in women with a high score (≥ 31); however, patients with HER2-positive disease and samples from the training cohort were included [9, 17]. In a more recent report from the prospective TAILORx study [16], patients with ER-positive, HER2-negative disease and a mid-range Oncotype Dx Recurrence score (11–25) did not have any chemotherapy benefit, and no significant interaction with treatment was observed [16]. Of note, results showed that women aged 50 years or younger with an intermediate Oncotype Dx risk score had a potential benefit from adjuvant chemotherapy. The MINDACT trial reported that women with a clinical high risk and molecular low risk had an excellent distant-recurrence risk after 5 years [8]. However, they were not able to address the question of chemotherapy benefit due to low power and low event rates.

Despite the prognostic ability of EPclin, it has not yet been shown whether it can predict chemotherapy benefit. Archival samples from previous randomised trials of endocrine therapy (ET) with or without chemotherapy (C) in patients with ER-positive, HER2-negative tumours have largely been exhausted. In addition, a prospective trial withholding chemotherapy from high-risk patients would be unethical. We therefore used an alternative study design to evaluate the ability of EPclin to predict adjuvant chemotherapy benefit for patients with ER-positive, HER2-negative disease. Here, we investigate in a non-randomised setting whether EPclin can predict chemotherapy benefit in pre- and postmenopausal women with early stage ER-positive, HER2-negative disease, who had received 5 years of endocrine therapy alone or in combination with chemotherapy.

## Methods

In this retrospective, comparative analysis, the EPclin was investigated in pre- and postmenopausal women with ER-positive, HER2-negative breast cancer treated with ET alone or ET + C using data from five large clinical trials. All patients from the GEICAM/9906 (*N* = 500) and GEICAM 2003/02 (*N* = 616) trials received ET + C. All patients from the ABCSG-6 (*N* = 378), ABCSG-8 (*N* = 1324), and TransATAC (*N* = 928) trials received five years of ET only, and served as our comparison group. The original ABCSG-6 trial included 2020 postmenopausal women with hormone receptor-positive breast cancer who received 5 years of tamoxifen alone or 5 years of tamoxifen plus aminoglutethimide for the first 2 years [18]. The ABCSG-8 trial randomised 3714 postmenopausal women with hormone receptor-positive breast cancer to 5 years of tamoxifen or 2 years of anastrozole followed by 3 years of tamoxifen [19]. Women in the TransATAC trial were postmenopausal and received 5 years of tamoxifen or anastrozole alone [20]. The GEICAM/9906 trial randomised pre- and postmenopausal women with lymph node-positive breast cancer to treatment with fluorouracil, epirubicin, and cyclophosphamide (FEC) or with FEC followed by weekly paclitaxel (FEC-P) [21]. Finally, the GEICAM 2002/03 trial entered pre- and postmenopausal women with node-negative disease and compared fluorouracil, doxorubicin, and cyclophosphamide (FAC) × 6 or FAC × 4 followed by wP × 8 (FAC-wP) [22].

The EPclin was developed in pre- and postmenopausal women with ER-positive, HER2-negative breast cancer [14]. For all five trials, EP molecular scores were generated by qRT-PCR gene analysis by Myriad Genetics, Inc. blinded to all clinical outcome data. The EP molecular score incorporates the expression of eight cancer-related genes (BIRC5, UBE2C, DHCR7, RBBP8, IL6ST, AZGP1, MGP, and STC2), three housekeeping genes (CALM2, OAZ1, and RPL37A), and one control gene (HBB). The EPclin incorporates nodal status and tumour size into the molecular score. Higher EPclin scores indicate a higher risk of distant recurrence. Predefined cut-off points were used to determine low- and high-risk patients (EPclin low risk < 3.3, EPclin high risk ≥ 3.3).

### Statistical analysis

The primary endpoint was distant recurrence-free interval (DRFI), defined as the time from randomisation in the primary study to distant recurrence of breast cancer. Local recurrence, regional recurrence, and contralateral second primary or secondary breast cancer in the ipsilateral breast were not considered as distant recurrence. Documented deaths due to breast cancer without distant recurrence occurring prior to death were also considered as a distant recurrence at the time of death. The secondary endpoint was breast cancer-free interval (BCFI), defined as the time from randomisation in the primary study to recurrence of any breast cancer (including local, regional, or distant recurrence or contralateral second primary or secondary breast cancer). Documented deaths due to breast cancer without recurrence of breast cancer or contralateral second primary breast cancer prior to the death were also considered as a recurrence of breast cancer at the time of death. All analyses were censored at 10 years of follow-up.

The primary objective of this analysis was to generate risk curves estimating 10-year DR as a continuous function of EPclin score separately for patients who received adjuvant ET + C and those who received ET only. Secondary objectives included (i) prognostic ability of EPclin for DRFI in ET + C and ET alone, (ii) prognostic ability of EPclin for BCFI in ET + C and ET alone, (iii) magnitude of benefit of ET + C compared to ET alone for DRFI and BCFI based on EPclin score, and (iv) as above but investigating EP molecular score. The primary analysis population were pre- and postmenopausal women with ER-positive, HER2-negative breast cancer who received five years of ET + C or ET alone. A predefined statistical analysis plan was approved by all research groups prior to data merge and data analyses. IS (TransATAC), DH (ABCSG), Lidija Soelkner (ABCSG), and Jesus Herranz Valera (GEICAM) had full access to clinical data for all five trials and performed all statistical analyses. We assessed 10-year distant recurrence risk using Cox proportional hazard models for a series of EPclin scores for ET alone and ET + C separately. To compare the prognostic performance of the continuous EPclin and EP scores, hazard ratios (HRs) and associated 95% confidence intervals (CIs) were estimated from Cox proportional hazards regression models. All HRs are for a one unit change in EPclin score. Multivariable models were adjusted for nodal status (0, 1–3, 4–10 and 10 + positive nodes), tumour size (T1a/b, T1c, T2 and T3), and tumour grade. A test for interaction was performed using Cox models containing chemotherapy treatment and EPclin score as a continuous variable. All statistical analyses were two-sided, and a *P* value of less than 0.05 was regarded as significant. All analyses were performed with Stata (version 13.1; StataCorp), R (version 3.3.2), and SAS software (version 9.4).

## Results

A total of 3746 pre- and postmenopausal women with ER-positive, HER2-negative breast cancer who received 5 years of ET and for whom EPclin was measured were included in this analysis. Table 1 shows baseline demographics by treatment group (ET alone vs. ET + C). In brief, 2630 women (70.2%) who received ET alone were all postmenopausal, significantly older, had significantly smaller tumours, significantly more node-negative disease, and significantly fewer poorly differentiated tumours (all *P *< 0.05) compared to those 1116 pre- and postmenopausal women who received ET + C (Table 1). 51% of patients who received ET + C were premenopausal. Women on ET alone had a significantly lower median EPclin score than those who received ET + C (3.07 vs. 3.67; *P *< 0.001) (Table 1, Supplemental Fig. 1).

**Table 1:** Patient demographics according to treatment group (ET alone vs. ET + C)

	ET only (*N* = 2630)	ET + C (*N* = 1116)	All (*N* = 3746)
Age (years), median (IQR)	63.7 (58.0–70.7)	51.0 (44.0–59.0)	61.00 (54.0–68.0)
Menopausal status			
Premenopausal	–	572 (51.3%)	572 (15.3%)
Postmenopausal	2630 (100.0%)	544 (48.8%)	3174 (84.7%)
Tumour stage			
T1a/b	422 (16.1%)	84 (7.5%)	506 (13.5%)
T1c	1333 (50.7%)	508 (45.5%)	1841 (49.2%)
T2	829 (31.5%)	487 (43.6%)	1316 (35.1%)
T3	43 (1.6%)	37 (3.3%)	80 (2.14%)
Unknown	3 (.1%)	–	3 (.08%)
Nodal status			
Negative	1846 (70.2%)	616 (55.2%)	2462 (65.7%)
1–3 positive	651 (24.8%)	326 (29.2%)	977 (26.1%)
4–10 positive	111 (4.2%)	139 (12.5%)	250 (6.7%)
10+ positive	22 (.8%)	35 (3.1%)	57 (1.5%)
Tumour grade			
Well	615 (23.4%)	131 (11.7%)	746 (19.9%)
Intermediate	1683 (64.0%)	605 (54.2%)	2288 (61.1%)
Poor	212 (8.1%)	322 (28.9%)	534 (14.3%)
Undetermined	120 (4.6%)	58 (5.2%)	178 (4.8%)
EP, median (IQR)	5.17 (3.88–6.77)	6.54 (4.81–8.66)	5.50 (4.11–7.36)
EPclin, median (IQR)	3.07 (2.54–3.67)	3.67 (3.05–4.45)	3.23 (2.66–3.94)
DR (0–10 years)	279 (10.6%)	146 (13.1%)	425 (11.4%)
Any recurrence (0–10 years)	398 (15.1%)	171 (15.3%)	569 (15.2%)
DR (5–10 years)	120/2202 (5.5%)	53/1008 (5.3%)	173/3210 (5.4%)
Any recurrence (5–10 years)	182/2155 (8.5%)	66/997 (6.6%)	248/3152 (7.9%)
Time to DRFI (years), median (IQR)	9.60 (5.97–10.00)	9.19 (7.47–10.00)	9.44 (6.75–10.00)
Time to BCFI (years), median (IQR)	9.38 (5.53–10.00)	9.12 (7.34–10.00)	9.29 (6.37–10.00)

*ET* endocrine therapy, *ET *+ *C* endocrine therapy plus chemotherapy, *IQR* interquartile range, *DR* distant recurrence, *DRFI* distant recurrence-free interval, *BCFI* breast cancer-free interval

Women receiving ET alone had a median follow-up time of 9.6 years (IQR 6–10). A total of 279 DR events (11%) were recorded for these women over a 10-year follow-up period, and 120 (6%; 120/2202) late DR events (years 5–10). Median time to DR for women on ET + C was 9 years (IQR 7–10) with a total of 146 (13%) DR events recorded, with 53 (5%; 53/1008) late DR events. Number of events and time to events are presented Table 1.

### Distant recurrence-free interval

**Fig. 1:**
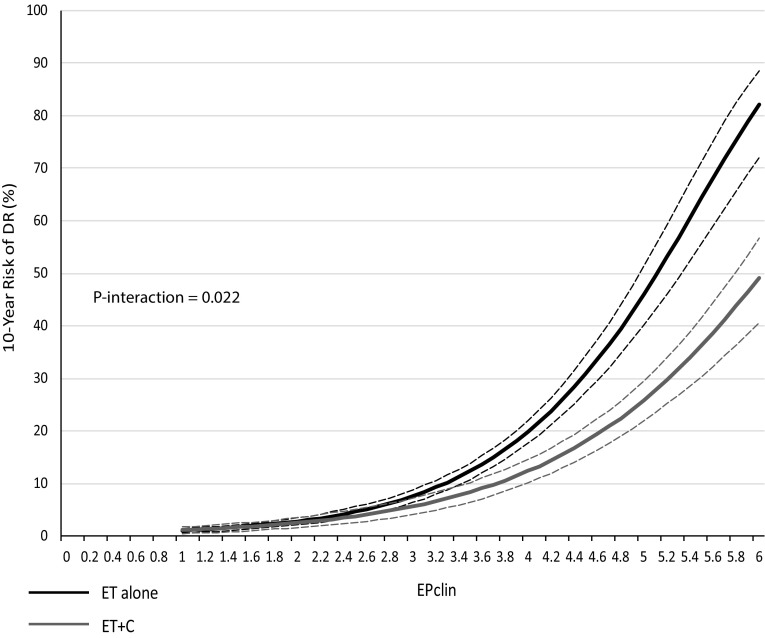
Likelihood of distant recurrence (DR) as a continuous function of EPclin for ET alone (black) and ET + C (grey) (dotted lines = 95% confidence intervals)

Figure 1 shows the relationship between EPclin scores and 10-year DR (%) for ET alone and ET + C. Women receiving ET alone had larger 10-year DR risks with increasing EPclin scores compared to those on ET + C (Table 2). For example, women on ET alone with an EPclin score of 5 had a 10-year DR risk of 46% compared to 26% for women who received ET + C, which translates to an absolute risk difference of 20%. In contrast, no differences in 10-year DR risks were observed for small EPclin scores, i.e. those who are at low risk of recurrence (Fig. 1, Table 2). We performed a sensitivity analysis excluding all premenopausal women and observed very similar results (EPclin score 5: 46% vs. 28% 10-year distant recurrence risk). To test the statistical strength between chemotherapy benefit and EPclin, an interaction test between EPclin as a continuous variable and treatment (ET vs. ET + C) was statistically significant (*P *= 0.022).

**Table 2:** 10-year risk (%) with 95% confidence intervals and absolute risk differences for distant recurrence for endocrine-treated patients alone (ET alone) and endocrine plus chemotherapy-treated patients (ET + C) according to EPclin score

EPclin score	ET alone	ET + C	Absolute risk difference between ET alone and ET + C
1	1.0% (0.6–1.4)	1.1% (0.5–1.7)	− 0.1%
2	2.8% (2.1–3.5)	2.5% (1.5–3.5)	.3%
3	7.6% (6.4–8.8)	5.7% (4.1–7.2)	1.9%
4	19.8% (17.6–22.0)	12.4% (10.1–14.6)	7.4%
5	46.1% (40.2–51.4)	25.8% (22.0–29.5)	20.3%
6	82.2% (72.1–88.6)	49.2% (40.5–56.7)	33.0%

*ET* endocrine therapy, *ET *+ *C* endocrine therapy plus chemotherapy, *EPclin* endopredict clinical

**Fig. 2:**
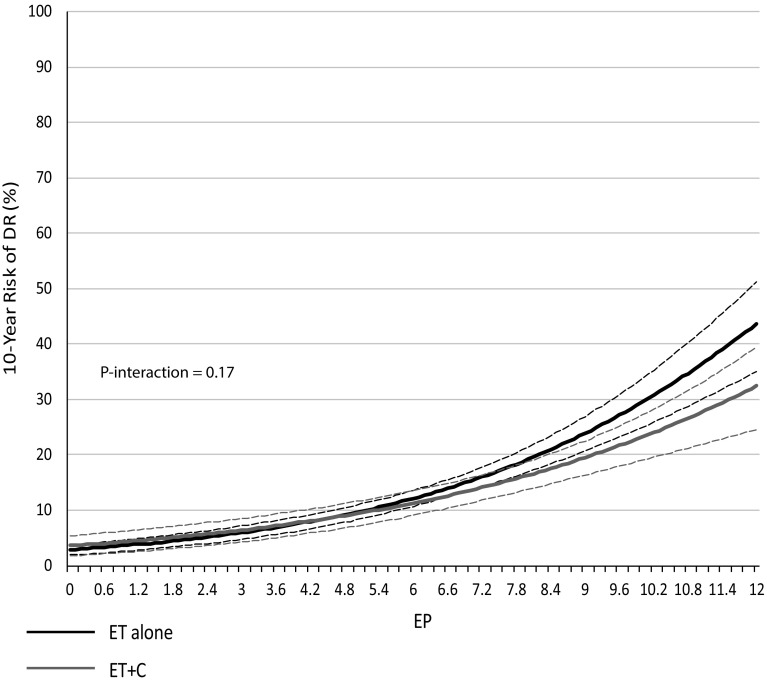
Likelihood of distant recurrence (DR) as a continuous function of EP for ET alone (black) and ET + C (grey) (dotted lines = 95% confidence intervals)

The molecular EP was separately investigated in both treatment groups. A separation of the 10-year DR risk curves between ET alone versus ET + C was observed with increasing EP scores but the difference was statistically not significant (Fig. 2), which was reflected with a non-significant interaction between treatment and EP score (*P *= 0.17) and overlapping 95% confidence intervals (Fig. 2). Still, EP was prognostic for DR in the ET alone group (HR 1.28 (1.23–1.34), *P *< 0.0001) and ET + C group (HR 1.22 (1.15–1.29), *P *< 0.0001), but these HRs are not directly comparable to those presented above for EPclin.

**Fig. 3:**
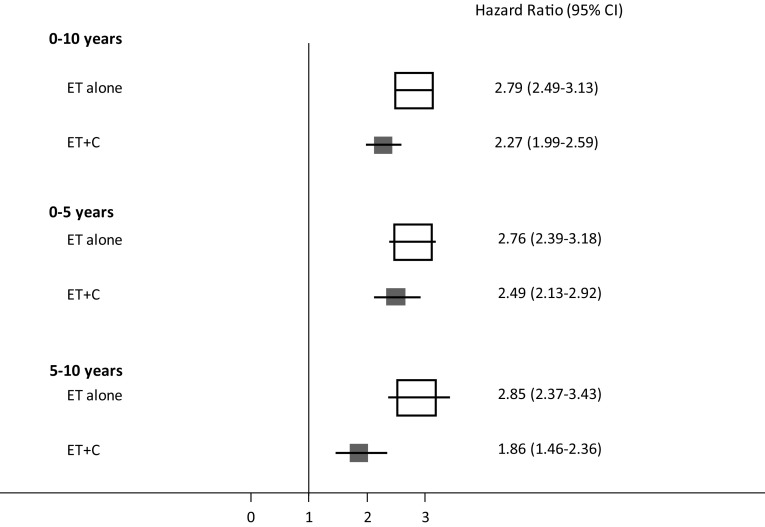
Univariable hazard ratios (95% CI) for the prognostic value of EPclin for DR according to treatment group and follow-up period

EPclin was highly prognostic in women who received ET alone (HR 2.79 (2.49–3.13), *P *< 0.0001) as well as in those who received ET + C (HR 2.27 (1.99–2.59), *P *< 0.0001) in a univariate analysis (Fig. 3). We furthermore investigated EPclin in two distinct follow-up periods; early (0–5 years) and late (5–10 years). EPclin was more prognostic in women who received ET alone compared to those on ET + C in both follow-up periods (Fig. 3). In women who received ET alone, EPclin was non-significantly more prognostic for late distant recurrence than for early distant recurrence (0–5 years: HR 2.76 vs. 5–10 years: HR 2.85; (Fig. 3)). In contrast, EPclin was non-significantly more prognostic for early distant recurrence in women who received ET + C (0–5 years: HR 2.49 vs. 5–10 years: HR 1.86). When adjusted for clinical parameters, EPclin remained highly prognostic in both treatment groups (data not shown).

### Breast cancer-free interval

**Fig. 4:**
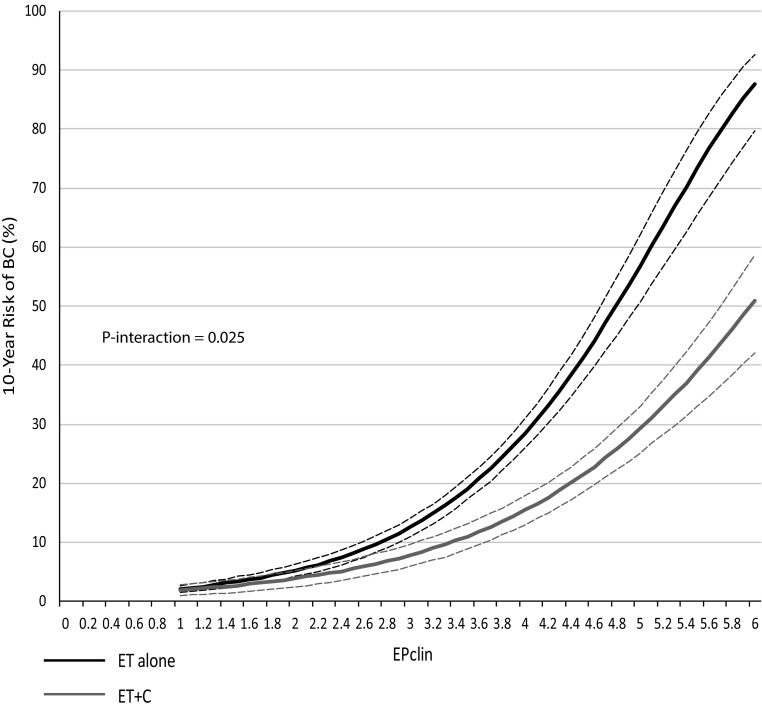
Likelihood of any recurrence (BC) as a continuous function of EPclin for ET alone (black) and ET + C (grey) (dotted lines = 95% confidence intervals)

Our secondary endpoint was BCFI, and we observed similar results. 10-year BC risks were significantly higher in women on ET alone with the increasing EPclin score (Fig. 4). Women on ET alone with an EPclin score of 5 had a 10-year recurrence risk of 57% compared to 29% for women who received ET + C. We observed a significant interaction between EPclin and treatment for any recurrence (*P *= 0.025). EPclin was highly prognostic for BC in women on ET alone (HR 2.50 (2.26–2.76), *P *< 0.0001) as well as in those who received ET + C (HR 2.06 (1.82–2.34), *P *< 0.0001). The prognostic value of EPclin remained significant after adjustments for clinical parameters. Finally, the EP molecular score was investigated for any recurrence, but no significant differences between EP scores and 10-year risks were observed (data not shown). A test for interaction was not significant between continuous EP score and treatment (*P *= 0.18). However, EP was significantly prognostic for any recurrence in women on ET alone (HR 1.25 (1.20–1.30), *P *< 0.0001) and those on ET + C (HR 1.19 (1.13–1.26), *P *< 0.0001).

## Discussion

Multigene tests are increasingly used for the prognostic evaluation of ER-positive, HER2-negative breast cancer and for the selection of patients for adjuvant chemotherapy. EPclin is highly prognostic in women with node-negative and node-positive disease [6, 11, 13, 14, 22]. Herein, we investigated the value of EPclin in women treated with endocrine therapy alone and those who received endocrine therapy plus chemotherapy. Patients with a high EPclin score (e.g. those considered high risk by EPclin) who received chemotherapy had a significantly lower 10-year distant recurrence risk than those who received endocrine therapy alone. Importantly, no differences in 10-year distant recurrence risks were observed between the two treatment groups for low EPclin scores (< 3.3). A significant test for interaction was observed, further emphasising the potential benefit of adding chemotherapy to those with high EPclin scores. Although our results were generated from a non-randomised, retrospective analysis, they are consistent with other studies that demonstrate chemotherapy benefit for patients with high-risk disease based upon molecular analyses [8, 9, 16]. The current analysis approach can facilitate insight into the predictive value of EPclin for women with ER-positive, HER2-negative breast cancer. Our results furthermore suggest that some women with a low EPclin score, but clinically high-risk tumours, received unnecessary chemotherapy. In addition, women with high EPclin scores who only received endocrine therapy alone would have been good candidates for adjuvant chemotherapy.

An initial report on the predictive value of Oncotype Dx Recurrence score showed the magnitude of chemotherapy benefit [9]. However, a proportion of women included in the study were also part of the discovery cohort for the Oncotype Dx Recurrence score, and some had HER2-positive disease. More recently, the TAILORx [16] trial reported that women with ER-positive, node-negative disease did not derive any chemotherapy benefit if they had a mid-range Oncotype Dx Recurrence score (< 25), indicating that only those categorised as high risk of developing a recurrence benefit from adjuvant chemotherapy. Our analysis of EPclin showed similar results as patients with a 10-year risk below 10% showed no substantial benefit from chemotherapy while those above 10% risk showed a chemotherapy benefit.

Most multigene prognostic test, including Oncotype Dx Recurrence score, Mammprint, Prosigna PAM50, are heavily weighted toward proliferation-related genes. The association of proliferation with response to chemotherapy varies based on the subgroup and is more prominent in higher proliferative subgroups (e.g. basal-like more than luminal A). Within each PAM50 subgroup, higher proliferative tumours were more likely to achieve complete pathological response [23]. In the neoadjuvant setting, 90% of multigene signatures and over 95% of individual genes that were significantly associated with response to chemotherapy can be attributed to proliferation [23]. EPclin incorporates proliferation-related genes, which might be one reason for our differential findings between the two treatment groups. Furthermore, EPclin incorporates nodal status and tumour size, both of which are strong prognostic factors for recurrence and both are traditionally considered important factors when deciding about adjuvant chemotherapy treatment. Incorporation of clinico-pathological factors allows a more accurate risk assessment than using a molecular signature alone [6]. Although in the Early Breast Cancer Trialists’ Collaborative Group overview [24] the relative benefit of polychemotherapy was similar in node-negative and node-positive breast cancer patients, and the absolute benefit of chemotherapy is much greater in women with node-positive disease, axillary lymph node involvement was not synonymous per se of a benefit from chemotherapy in patients with ER-positive, HER2-negative disease.

Strengths of our analysis include a large group of 3746 pre- and postmenopausal women with ER-positive, HER2-negative breast cancer from large randomised clinical trials with long-term follow-up. Furthermore, we have clinical outcome data for all patients and used well characterised tissue samples. EPclin for all trials was measured in the same laboratory, with all personnel being blinded to clinical outcome data. We included patients who have been treated with modern chemotherapy regimens such as FEC/FAC with or without paclitaxel. Limitations include that we were unable to compare the predictive value of EPclin in a prospective, randomised trial design. We used an indirect approach investigating the value of EPclin in women from large clinical trials who received endocrine therapy alone and compared them to those who received endocrine therapy plus chemotherapy. We believe that this retrospective approach is an effective way to evaluate the clinical usefulness of EPclin as data from large prospective, randomised trials are not available due to ethical, time, and resource considerations. Furthermore, EPclin incorporates nodal status and tumour size into its molecular score and therefore largely accounts for the two differential patient cohorts with different baseline risks in our analysis. This might also be the reason why no significant differences in 10-year distant recurrence risk was observed between ET alone versus ET + C with increasing molecular EP score. Without accounting for tumour size and nodal status, the absolute recurrence risk at a specific molecular score value depends on the baseline risk, which is higher in patient with node-positive disease.

In summary, EPclin was highly prognostic in women with ER-positive, HER2-negative breast cancer who received endocrine therapy alone and in those who received chemotherapy plus endocrine therapy. The results highlight that many women with low EPclin scores were likely to have been “over-treated” with chemotherapy and that many women with high EPclin scores may not have received necessary chemotherapy treatment. Overall, our analysis suggests that women with high EPclin scores benefitted from chemotherapy compared to those with the same EPclin score but receiving endocrine therapy alone, irrespective of node-positivity of the disease. Although our approach was an indirect comparison of EPclin in chemotherapy plus endocrine therapy treated women versus endocrine therapy alone, we demonstrated that a high EPclin sore can predict chemotherapy benefit in women with ER-positive, HER2-negative disease.

## Electronic supplementary material

Below is the link to the electronic supplementary material.

Supplementary material 1 (EPS 1121 kb)

Supplementary material 2 (DOCX 13 kb)

Supplementary material 3 (DOCX 13 kb)
