# Supplementary material for: Prediction of chemotherapy benefit by EndoPredict in patients with breast cancer who received adjuvant endocrine therapy plus chemotherapy or endocrine therapy alone
Source: Breast Cancer Res Treat. 2019 Apr 30;176(2):377–86. doi: 10.1007/s10549-019-05226-8 (PMC6555778; doi:10.1007/s10549-019-05226-8)
Supplement: Supplementary file 2 — Supplementary material 2 (DOCX 13 kb) [file 10549_2019_5226_MOESM2_ESM.docx]

**Supplemental Table 1**: 10-year risk (%) with 95% confidence intervals and absolute risk differences for distant recurrence for endocrine treated patients alone (ET alone) and endocrine plus chemotherapy treated patients (ET+C) according to molecular EP score.

| **EP score** | **ET alone** | **ET+C** | **Absolute risk difference between ET alone and ET+C** |
| --- | --- | --- | --- |
| 1 | 3.7% (2.6-4.7) | 4.4% (2.4-6.3) | -0.7% |
| 2 | 4.7% (3.6-5.8) | 5.3% (3.3-7.3) | -0.6% |
| 3 | 6.0% (4.8-7.2) | 6.4% (4.3-8.5) | -0.4% |
| 4 | 7.6% (6.3-8.8) | 7.8% (5.6-9.9) | -0.2% |
| 5 | 9.6% (8.3-10.9) | 9.4% (7.1-11.6) | 0.2% |
| 6 | 12.1% (10.7-13.6) | 11.3% (9.1-13.5) | 0.8% |
| 7 | 15.3% (13.6-17.0) | 13.6% (11.3-15.8) | 1.7% |
| 8 | 19.2% (16.9-21.4) | 16.3% (13.8-18.8) | 2.9% |
| 9 | 23.9% (20.7-26.9) | 19.5% (16.4-22.5) | 4.4% |
| 10 | 29.5% (25.0-33.8) | 23.2% (19.0-27.2) | 6.3% |
| 11 | 36.1% (29.8-41.9) | 27.5% (21.8-32.8) | 8.6% |
