# Supplementary material for: Prediction of chemotherapy benefit by EndoPredict in patients with breast cancer who received adjuvant endocrine therapy plus chemotherapy or endocrine therapy alone
Source: Breast Cancer Res Treat. 2019 Apr 30;176(2):377–86. doi: 10.1007/s10549-019-05226-8 (PMC6555778; doi:10.1007/s10549-019-05226-8)
Supplement: Supplementary file 3 — Supplementary material 3 (DOCX 13 kb) [file 10549_2019_5226_MOESM3_ESM.docx]

**Supplemental Table 2**: Multivariable hazard ratios (95% CI) for the prognostic value of the continuous molecular EP for DR according to treatment group.

|  | **ET only (N=2506)** | | | **ET&C (N=1058)** | | |
| --- | --- | --- | --- | --- | --- | --- |
|  | **HR (95% CI)** | **P-value** | **HR (95% CI)** | | **P-value** |  |
| EP score | 1.20 (1.14-1.26) | <0.0001 | 1.17 (1.09-1.25) | | <0.0001 |  |
| N0 | Reference | - | Reference | | - |  |
| N1-3 | 1.92 (1.46-2.53) | <0.0001 | 4.10 (2.55-6.59) | | <0.0001 |  |
| N4-10 | 4.70 (3.22-6.88) | <0.0001 | 6.40 (3.87-10.61) | | <0.0001 |  |
| N10+ | 19.08 (11.04-32.99) | <0.0001 | 11.26 (5.71-22.21) | | <0.0001 |  |
| Low grade | Reference | - | Reference | | - |  |
| Intermediate grade | 1.81 (1.20-2.73) | 0.005 | 1.58 (0.84-2.96) | | 0.152 |  |
| High grade | 2.09 (1.26-3.46) | 0.004 | 1.38 (0.70-2.75) | | 0.355 |  |
| T1a/b | Reference | - | Reference | | - |  |
| T1c | 2.00 (1.12-3.57) | 0.019 | 1.62 (0.58-4.53) | | 0.362 |  |
| T2 | 3.53 (1.98-6.30) | <0.0001 | 2.52 (0.91-6.95) | | 0.074 |  |
| T3 | 2.59 (1.08-6.23) | 0.033 | 2.63 (0.73-9.39) | | 0.138 |  |
